# Metabolic-dimension CKM staging and cardiometabolic components predict incident MASLD: a longitudinal community-based cohort study

**DOI:** 10.3389/fendo.2026.1847134

**Published:** 2026-05-22

**Authors:** Xinyang Long, Meihui Wu, YeMei Mo, Yingqi Zhang, Yining Lin, Qiuling Zhang, Zengnan Mo

**Affiliations:** 1 School of Public Health, Guangxi Medical University, Nanning, Guangxi, China; 2 Centre for Genomic and Personalized Medicine, Guangxi Key Laboratory for Genomic and Personalized Medicine, Guangxi Collaborative Innovation Centre for Genomic and Personalized Medicine, Guangxi Medical University, Nanning, Guangxi, China; 3 Department of Nursing, Daping Hospital, Army Medical University, Chongqing, China; 4 The First People’s Hospital of Yulin, Yulin, Guangxi, China; 5 School of Basic Medical College, Guangxi Medical University, Nanning, Guangxi, China; 6 Institute of Urology and Nephrology, First Affiliated Hospital of Guangxi Medical University, Guangxi Medical University, Nanning, Guangxi, China

**Keywords:** cardiometabolic risk, cardiovascular endocrinology, cardiovascular-kidney-metabolic syndrome, Chinese population, CKM staging, community cohort, hepatology, longitudinal study

## Abstract

**Background:**

Metabolic dysfunction-associated steatotic liver disease (MASLD) shares metabolic pathways with cardiovascular-kidney-metabolic (CKM) syndrome, but prospective community-based evidence linking CKM metabolic risk burden to incident MASLD remains limited.

**Methods:**

We analysed longitudinal health examination data from a community cohort in Guangxi, southern China. Because subclinical cardiovascular and kidney assessments were unavailable, CKM staging was operationalised as a metabolic-dimension staging system based on five cardiometabolic (CM) components. Cross-sectional analyses included 3,315 participants. The primary forward cohort included 2,055 participants free of MASLD at baseline, with 294 incident events over a median 2.61 years. An exploratory reverse cohort included 648 participants at CKM early stage, with 407 progression events over a median 1.77 years. Ultrasonographic fatty liver alone was used as a sensitivity outcome to address definitional overlap between MASLD and CM components.

**Results:**

Each additional CM component was associated with higher odds of prevalent MASLD (OR = 1.94, 95% CI: 1.79–2.10), with a similar estimate for fatty liver alone (OR = 1.90, 95% CI: 1.75–2.05). In the forward cohort, metabolic-dimension CKM Stage 2 was associated with higher incident MASLD risk compared with Stage 0 (HR = 2.33, 95% CI: 1.20–4.54), and the fatty-liver-alone sensitivity outcome yielded a consistent estimate (HR = 2.95, 95% CI: 1.39–6.26). Among individual CM components, excess adiposity was the strongest predictor (mutually adjusted HR = 2.97, 95% CI: 2.00–4.41), followed by hypertriglyceridaemia (HR = 1.63, 95% CI: 1.23–2.18). In the exploratory reverse cohort, baseline MASLD was associated with CKM stage progression in the age- and sex-adjusted model (HR = 2.13, 95% CI: 1.49–3.03), but this association attenuated after adjustment for baseline BMI (HR = 1.18, 95% CI: 0.81–1.71).

**Conclusion:**

Metabolic-dimension CKM staging and CM component burden predicted incident MASLD, with excess adiposity and **hypertriglyceridaemia** as dominant component-level predictors. The forward association was robust to removing MASLD-CM definitional overlap. In contrast, baseline MASLD was not clearly associated with CKM stage progression after accounting for baseline adiposity and related metabolic severity.

## Introduction

Metabolic dysfunction-associated steatotic liver disease (MASLD) is the most common chronic liver condition worldwide, affecting approximately 30% of adults globally and a higher proportion in Asia-Pacific populations (1, 2). In 2023, a multi-society Delphi consensus replaced the term non-alcoholic fatty liver disease (NAFLD) with MASLD, emphasizing the central role of metabolic dysfunction in disease pathogenesis (3). Population-based studies from China have reported MASLD prevalence exceeding 25%, with clustering in regions undergoing rapid nutritional transition (4, 5).

In the same year, the American Heart Association (AHA) introduced the cardiovascular-kidney-metabolic (CKM) syndrome framework (6). This staging system (Stages 0–4) integrates cardiovascular disease, chronic kidney disease, and metabolic risk factors—including excess adiposity, type 2 diabetes, and metabolic syndrome—into a single pathophysiological model. The framework encourages integrated risk assessment rather than organ-specific management. However, the current CKM staging system does not include liver disease, despite the liver’s involvement in lipoprotein metabolism, glucose homeostasis, and inflammatory signalling.

MASLD and CKM syndrome share several pathophysiological mechanisms, including insulin resistance, low-grade inflammation, oxidative stress, and endothelial dysfunction (7, 8). The liver is not only affected by cardiometabolic insults but also propagates systemic metabolic dysfunction through hepatokine secretion and dysregulated lipogenesis. On this basis, Zhou et al. proposed expanding the CKM framework to include the liver, forming a cardiovascular-kidney-liver-metabolic (CKLM) concept (9). Several recent studies have linked CKM staging to MASLD prevalence, liver fibrosis risk, and mortality (10–12), and the co-rising burden of both conditions in Asia has been highlighted (13). However, two questions are unresolved. First, no prospective community-based study has evaluated whether CKM staging predicts incident MASLD. Second, the independent longitudinal contributions of individual cardiometabolic components to MASLD risk have not been disentangled in a mutually adjusted framework.

Using longitudinal health examination data from a community population in Guangxi, southern China, the primary aim of this study was to evaluate whether *metabolic-dimension* CKM staging (Stages 0–2, based on the metabolic risk domain of the AHA framework) and individual cardiometabolic components predict incident MASLD. Routine community health examinations in this cohort did not include subclinical cardiovascular or kidney imaging, precluding application of the full AHA CKM framework (Stages 3–4); accordingly, our operationalisation is limited to the metabolic risk dimension. As a secondary, exploratory analysis — and with the caveat that baseline MASLD is structurally closer to the ≥2-CM outcome because MASLD requires ≥1 CM component at baseline — we also examined whether baseline MASLD was associated with subsequent CKM stage progression.

## Materials and methods

### Study design and participants

This study used longitudinal health examination data from the Wuliqiao Community Health Service Centre, affiliated with the First People’s Hospital of Yulin, Guangxi Zhuang Autonomous Region, southern China. Health examinations were conducted between 2020 and 2025 as part of China’s National Basic Public Health Services (BPHS) program, which provides standardized annual or biennial assessments to registered community residents (14). At each visit, participants underwent anthropometric measurements, biochemical testing, abdominal ultrasonography, and structured health questionnaires.

**Figure 1:**
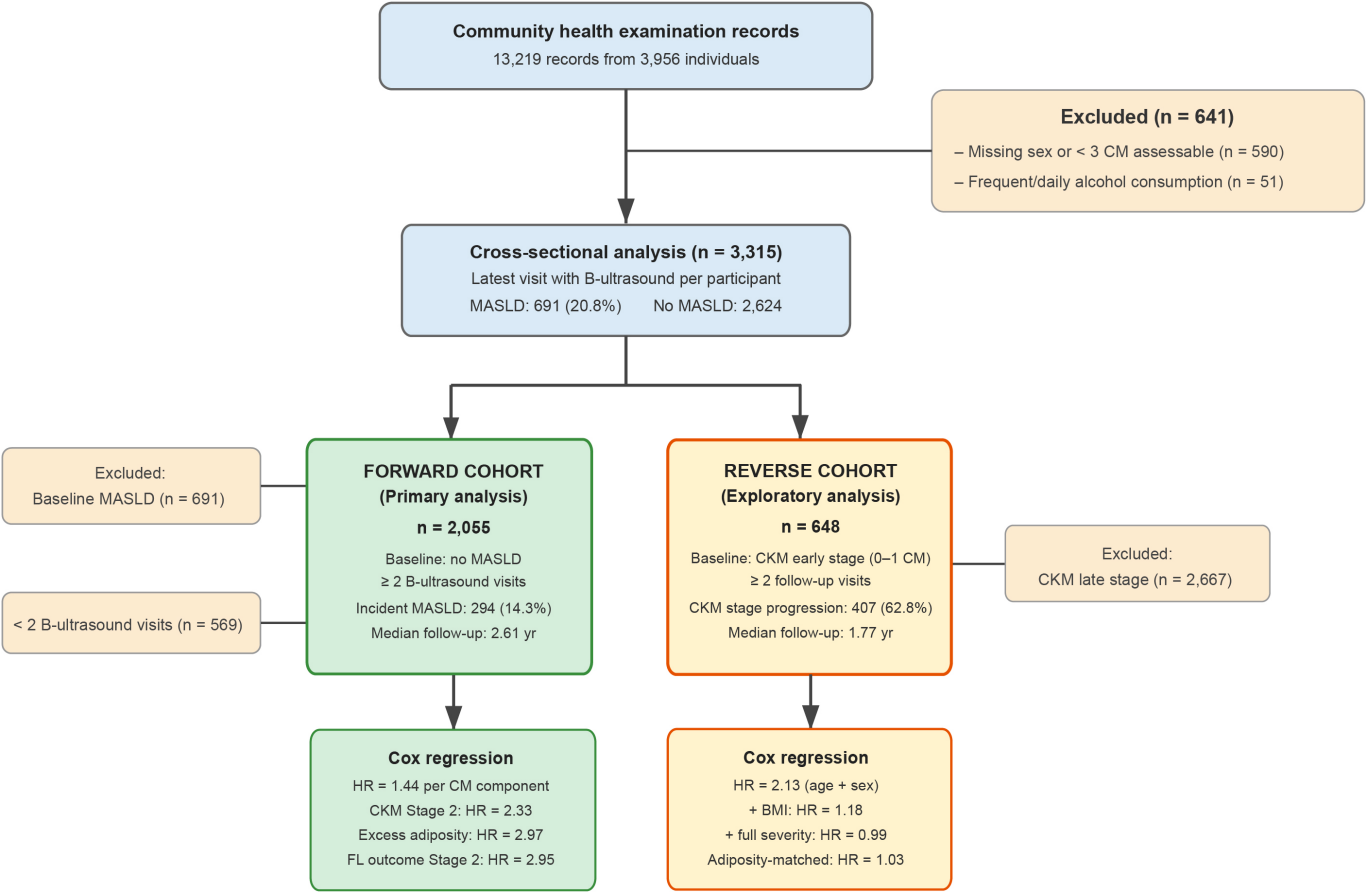
Study flow diagram. Three analytic datasets were constructed from the community health examination cohort. Cross-sectional dataset: n = 3,315. Forward cohort (primary analysis): n = 2,055 participants free of MASLD at baseline, 294 incident events. Reverse cohort (exploratory): n = 648 participants at metabolic-dimension CKM early stage (0–1 CM components) at baseline, 407 events of progression to CKM late stage. MASLD was defined as ultrasonographic hepatic steatosis plus at least one cardiometabolic risk component; ultrasonographic fatty liver alone (no metabolic requirement) was retained as a pre-specified sensitivity exposure/outcome.

A total of 3,956 unique individuals (13,219 examination records) were identified. After excluding participants with missing sex data or fewer than three assessable CM components (n = 590) and those reporting frequent or daily alcohol consumption (n = 51), we constructed three analytic datasets from the remaining 3,315 individuals (**Figure 1**). The cross-sectional dataset included 3,315 participants with at least three of five CM components assessable and an ultrasonographic fatty liver evaluation. The forward cohort included 2,055 participants free of MASLD at baseline who had at least one follow-up ultrasound examination. The exploratory reverse cohort included 648 participants classified as CKM early stage (0–1 CM components) at baseline with at least one follow-up visit; this analysis examined whether baseline MASLD was associated with subsequent progression to CKM late stage (≥2 CM components), with explicit acknowledgement of the structural limitations described in the Statistical Analysis section. Participants who reported frequent or daily alcohol consumption were excluded.

Self-reported history of physician-diagnosed hypertension and type 2 diabetes, together with the use of antihypertensive and glucose-lowering medications, was available from the baseline community questionnaire for a subset of participants (n = 1,849 with linkable records) and was used in pre-specified medication sensitivity analyses. Lipid-lowering medication use was not systematically recorded.

This study was approved by the relevant institutional ethics committees (see Ethics Statement section). All participants provided written informed consent before participation.

### Variable definitions

We defined five cardiometabolic (CM) risk components based on measured values at each examination: (1) excess adiposity, BMI ≥23 kg/m² or waist circumference ≥90 cm for men/≥80 cm for women (Asia-Pacific cutoffs) (15); (2) hyperglycaemia, fasting blood glucose (FBG) ≥5.6 mmol/L; (3) elevated blood pressure, systolic BP ≥130 mmHg or diastolic BP ≥85 mmHg; (4) hypertriglyceridaemia, serum triglycerides (TG) ≥1.7 mmol/L; and (5) low HDL cholesterol, HDL-C <1.0 mmol/L for men and <1.3 mmol/L for women. The total number of CM components present (0–5) was calculated for each participant. Missing components were treated as absent, which may underestimate the true cardiometabolic burden and bias associations toward the null; the complete-case sensitivity analysis (restricted to participants with all five components assessable) yielded consistent results. Participants with fewer than three assessable components were excluded.

Fatty liver was diagnosed by experienced sonographers using abdominal ultrasonography with standardized criteria: hepatorenal echo contrast, liver brightness, deep beam attenuation, and vascular blurring (16). MASLD was defined as ultrasonographic hepatic steatosis plus at least one cardiometabolic risk component (3), consistent with the 2023 multi-society Delphi consensus. Ultrasonographic fatty liver alone (without requiring metabolic criteria) was retained as a sensitivity outcome.

We operationalised *metabolic-dimension* CKM staging based on the metabolic risk domain of the AHA 2023 CKM framework (6), using the following three levels: Stage 0, no cardiometabolic risk factors; Stage 1, excess adiposity or a single metabolic abnormality; Stage 2, metabolic risk factor clustering (≥2 CM components). Because routine community health examinations did not include echocardiography, coronary imaging, or measurement of urinary albumin-to-creatinine ratio sufficient to ascertain subclinical cardiovascular or kidney disease, the full AHA Stages 3 (subclinical CVD/CKD) and 4 (clinical CVD/CKD) could not be applied; our staging therefore reflects only the metabolic dimension. We use the term “metabolic-dimension CKM staging” throughout to make this restriction explicit. This operationalisation is consistent with prior community-based studies that also restricted CKM staging to the metabolic domain because of similar data constraints (10 ,12). For the exploratory reverse cohort analysis, CKM early stage was defined as Stage 0–1 and CKM late stage as Stage 2 (≥2 CM components).

The triglyceride-glucose (TyG) index was computed as ln[TG (mg/dL) × FBG (mg/dL)/2], with TG and FBG values converted from mmol/L to mg/dL using the factors 88.57 and 18.02, respectively (17). Metabolic syndrome (MetS) was defined according to the 2013 Chinese Diabetes Society (CDS) criteria (18): central obesity (WC ≥90 cm men/≥85 cm women), elevated BP (≥130/85 mmHg), hyperglycaemia (FBG ≥6.1 mmol/L), hypertriglyceridaemia (TG ≥1.7 mmol/L), and low HDL-C (<1.04 mmol/L men/<1.30 mmol/L women); MetS was diagnosed when three or more criteria were met. The CDS MetS definition uses a female waist-circumference cutoff of ≥85 cm, whereas the CKM adiposity component used the Asia-Pacific cutoff of ≥80 cm; therefore, MetS and individual CM components were analysed as separate guideline-based variables.

### Statistical analysis

Continuous variables with normal distributions were expressed as mean ± standard deviation (SD); non-normally distributed variables were reported as median (interquartile range [IQR]). Baseline characteristics (**Table 1**) are compared using standardised mean differences (SMD), with an absolute value >0.1 considered to indicate meaningful imbalance; P values were omitted from **Table 1** because with n = 3,315 even negligible between-group differences reach conventional significance and are therefore uninformative for balance assessment.

**Table 1:** Baseline characteristics of cross-sectional study participants stratified by MASLD status (n = 3,315).

Characteristic	Overall (n = 3,315)	No MASLD (n = 2,624)	MASLD (n = 691)	SMD
Age, years, mean ± SD	69.51 ± 10.69	68.88 ± 11.30	71.93 ± 7.47	0.32
Female, n (%)	1,903 (57.4)	1,468 (55.9)	435 (63.0)	0.14
BMI, kg/m², mean ± SD	23.88 ± 3.20	23.44 ± 3.10	25.55 ± 3.02	0.69
WC, cm, mean ± SD	86.42 ± 8.84	85.25 ± 8.66	90.86 ± 8.08	0.67
SBP, mmHg, mean ± SD	134.39 ± 15.43	133.47 ± 15.28	137.88 ± 15.54	0.29
DBP, mmHg, mean ± SD	80.69 ± 9.01	80.28 ± 9.06	82.22 ± 8.67	0.22
FBG, mmol/L, median [IQR]	4.90 [4.30, 5.79]	4.84 [4.26, 5.65]	5.12 [4.44, 6.37]	0.26
TG, mmol/L, median [IQR]	1.32 [0.99, 1.82]	1.25 [0.93, 1.67]	1.62 [1.25, 2.33]	0.26
HDL-C, mmol/L, mean ± SD	1.35 ± 0.41	1.37 ± 0.41	1.26 ± 0.41	0.27
TyG index, median [IQR]	8.57 [8.22, 8.95]	8.49 [8.16, 8.84]	8.89 [8.50, 9.29]	0.66
CM components, mean ± SD	2.30 ± 1.20	2.11 ± 1.15	3.02 ± 1.11	0.80
MetS, n (%)	1,021 (30.8)	628 (23.9)	393 (56.9)	0.71
Metabolic-dimension CKM Stage 0, n (%)	196 (5.9)	196 (7.5)	0 (0.0)	—
Metabolic-dimension CKM Stage 1, n (%)	644 (19.4)	588 (22.4)	56 (8.1)	—
Metabolic-dimension CKM Stage 2, n (%)	2,475 (74.7)	1,840 (70.1)	635 (91.9)	—
Excess adiposity, n (%)	2,486 (75.0)	1,837 (70.0)	649 (93.9)	0.65
hyperglycaemia, n (%)	980 (29.6)	718 (27.4)	262 (38.0)	0.23
Elevated BP, n (%)	2,256 (68.3)	1,718 (65.8)	538 (78.0)	0.27
hypertriglyceridaemia, n (%)	890 (28.9)	575 (24.1)	315 (45.7)	0.47
Low HDL-C, n (%)	1,007 (34.1)	686 (30.3)	321 (46.5)	0.34

Values are median (IQR) or n (%). Baseline balance is reported using standardised mean differences (SMDs); SMD > 0.1 indicates meaningful imbalance. Metabolic-dimension CKM stages were defined by the number of cardiometabolic (CM) components: Stage 0, 0; Stage 1, 1; and Stage 2, ≥2. MASLD is absent in Stage 0 by definition, because MASLD requires at least one CM component. Percentages for binary CM components were calculated among participants with available data; TG was assessable in 3,080 participants (92.9%) and HDL-C in 2,951 (89.0%). MASLD, metabolic dysfunction-associated steatotic liver disease; CM, cardiometabolic; SMD, standardised mean difference; IQR, interquartile range; TyG, triglyceride-glucose index.

The cross-sectional analysis established the dose–response relationship between CM component count and MASLD. Logistic regression was adjusted for age and sex.

The forward cohort comprised the primary analysis. Cox proportional hazards models estimated hazard ratios (HR) and 95% confidence intervals (CI) for incident MASLD. Two models were fitted: Model 1 adjusted for age and sex; Model 2 additionally adjusted for smoking status (never/former/current) and drinking status (never/occasional). Predictors were evaluated in three tiers: (1) metabolic-dimension CKM staging (Stage 0–2, categorical and per-stage trend); (2) individual CM components, each examined separately and in a mutually adjusted model; and (3) CM component count (continuous), MetS status, and TyG index quartiles. Because CKM staging and CM component count are defined by the same metabolic variables (excess adiposity, blood glucose, blood pressure, triglycerides, HDL-C), these parameters were not included as additional covariates; adjusting for them would constitute over-adjustment by conditioning on components of the exposure. We note that the five CM components are physiologically collinear and partially on a shared causal pathway (adiposity → dyslipidaemia/hyperglycaemia → hepatic steatosis); mutually adjusted component-level estimates therefore represent partial (conditional) rather than independent causal effects. Follow-up time was calculated from the first examination to the date of MASLD diagnosis (for events) or the last examination (for censored participants). The proportional hazards assumption was tested using Schoenfeld residuals. Kaplan–Meier curves were compared using log-rank tests. Subgroup analyses were performed by sex, age (<65 vs. ≥65 years), and BMI (<23 vs. ≥23 kg/m²).

For the exploratory reverse cohort, Cox regression evaluated the association between baseline MASLD and progression from CKM early stage to CKM late stage. Because MASLD-positive participants start closer to the ≥2-CM outcome, we used a staged attenuation sequence to isolate the contribution of baseline adiposity and metabolic severity. To quantify the contribution of this structural imbalance, we employed a pre-specified stepwise attenuation sequence. Model 1 adjusted for age and sex; Model 2 additionally incorporated baseline body mass index (BMI) as a continuous variable; and Model 3 further adjusted for a comprehensive set of continuous metabolic severity markers (BMI, waist circumference, fasting glucose, triglycerides, HDL-C, and systolic blood pressure). This incremental approach was designed to isolate the contribution of specific metabolic dimensions and to mitigate multicollinearity. To further align baseline clinical risk between groups, two additional strategies were used: adjustment for baseline CM component count, and a restricted analysis within the CM = 1 stratum. Within this stratum, standardized mean differences (SMDs) were calculated to quantify the baseline imbalance between MASLD-positive and MASLD-negative participants.

Sensitivity analyses included: (1) a 12-month washout excluding early events; (2) ultrasonographic fatty liver alone (no metabolic requirement) as an alternative outcome (cross-sectional and forward cohort) or exposure (reverse cohort), which removes the definitional overlap between CM components and MASLD; (3) complete-case analysis restricted to participants with all five CM components assessable; (4) a medication-use sensitivity analysis for the blood pressure component, in which the association between elevated blood pressure and incident MASLD was re-estimated after excluding participants on antihypertensive medication, with continuous systolic blood pressure (per 10 mmHg) as an alternative exposure in the medication-naive subset. All analyses were performed using R version 4.5.2. Two-sided P values <0.05 were considered statistically significant.

## Results

### Baseline characteristics

The cross-sectional analysis included 3,315 community residents (mean age 69.5 ± 10.7 years; 57.4% female). The overall prevalence of MASLD was 20.8% (691/3,315). Compared with participants without MASLD, those with MASLD had higher BMI (25.6 ± 3.0 vs. 23.4 ± 3.1 kg/m²), waist circumference (90.9 ± 8.1 vs. 85.2 ± 8.7 cm), fasting glucose (median 5.12 vs. 4.84 mmol/L), triglycerides (median 1.62 vs. 1.25 mmol/L), and lower HDL-C (1.26 ± 0.41 vs. 1.37 ± 0.41 mmol/L; all P < 0.001). MetS was present in 56.9% of participants with MASLD versus 23.9% of those without (*P* < 0.001). In the CKM staging distribution, 5.9% were Stage 0, 19.4% Stage 1, and 74.7% Stage 2, reflecting the high cardiometabolic burden in this community population (**Table 1**).

### Cross-sectional analysis: dose–response relationship

MASLD prevalence increased monotonically with the number of CM components: 0% (0 components), 8.7% (1), 16.1% (2), 27.5% (3), 39.2% (4), and 53.0% (5) (P for trend < 0.001; **Supplementary Table 1**). The zero prevalence at 0 components is consistent with the MASLD definition requiring at least one cardiometabolic criterion. After adjustment for age and sex, each additional CM component was associated with 94% higher odds of MASLD (OR = 1.94, 95% CI: 1.79–2.10; P < 0.001). This gradient motivated the detailed analysis in the forward cohort.

### Forward cohort: CKM staging and components predict incident MASLD

The forward cohort comprised 2,055 participants without MASLD at baseline. Over a median follow-up of 2.61 years (IQR: 1.71–3.77), 294 participants developed incident MASLD (cumulative incidence 14.3%).

#### CKM staging

**Figure 2:**
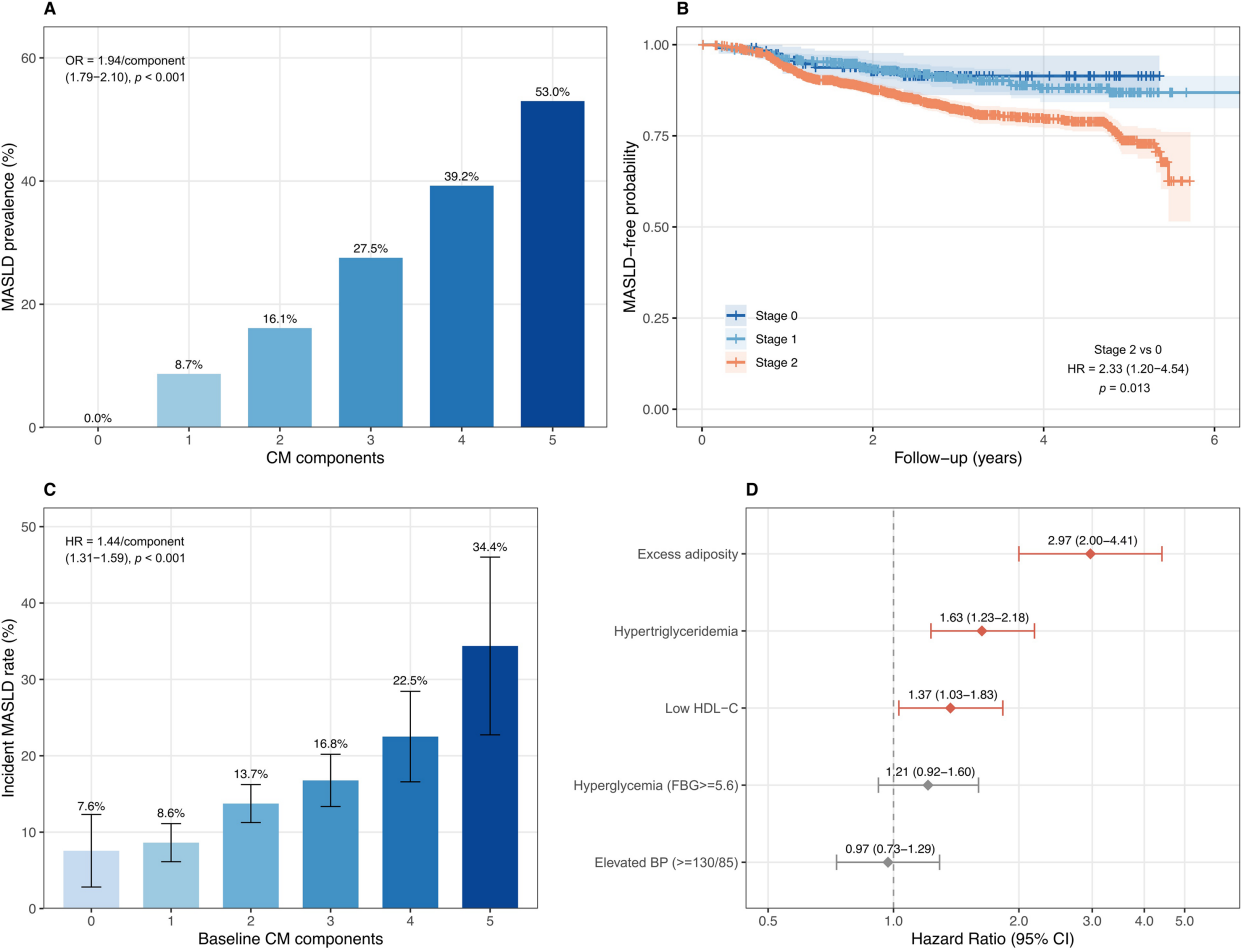
Forward cohort: metabolic-dimension CKM staging and components predict incident MASLD (n = 2,055; 294 events). **(A)** Cross-sectional MASLD prevalence by number of cardiometabolic (CM) components. **(B)** Kaplan–Meier curves for incident MASLD by baseline metabolic-dimension CKM stage. **(C)** Cumulative incidence of MASLD by baseline CM component count. **(D)** Forest plot of individual CM component hazard ratios from the mutually adjusted Model 2. Full estimates are reported in **Table 2**; the blood pressure medication sensitivity analysis is reported in **Supplementary Table 2**.

At baseline, 119 participants (5.8%) were metabolic-dimension CKM Stage 0, 487 (23.7%) Stage 1, and 1,449 (70.5%) Stage 2. MASLD incidence rates increased across stages: 2.74, 3.09, and 6.18 per 100 person-years for Stages 0, 1, and 2, respectively. In the age- and sex-adjusted model, Stage 2 was associated with a higher risk of incident MASLD than Stage 0 (HR 2.33, 95% CI 1.20–4.54; *P* = 0.013). The estimate for Stage 1 was imprecise and not statistically significant (HR 1.14, 95% CI 0.56–2.35; *P* = 0.716), likely reflecting the small Stage 0 reference group (n = 119; 9 events). The per-stage trend was significant (HR = 1.80 per stage, 95% CI: 1.39–2.31, P < 0.001). Results were essentially unchanged in Model 2 (additionally adjusted for smoking and drinking: Stage 2 HR = 2.37, 95% CI: 1.22–4.62; **Table 2**). Kaplan–Meier analysis showed progressive separation among CKM stages (log-rank P < 0.001; **Figure 2B**).

**Table 2:** Cox proportional hazards regression for incident MASLD in the forward cohort (n = 2,055; 294 events; median follow-up 2.61 years).

Panel A. Metabolic-dimension CKM staging and CM component count						
Predictor	Events/N		Model 1 HR (95% CI)			Model 2 HR (95% CI)
Metabolic-dimension CKM Stage 0 (ref)	9/119		1.00			1.00
Metabolic-dimension CKM Stage 1 vs 0	42/487		1.14 (0.56–2.35)			1.16 (0.57–2.39)
Metabolic-dimension CKM Stage 2 vs 0	243/1,449		2.33 (1.20–4.54)			2.37 (1.22–4.62)
Per stage (trend)	294/2,055		1.80 (1.39–2.31)			1.80 (1.40–2.32)
CM count (per 1)	294/2,055		1.44 (1.31–1.59)			1.45 (1.32–1.60)
MetS (yes vs no)	—		2.09 (1.64–2.64)			2.12 (1.67–2.69)
Panel B. Individual CM components						
Component	Model 1 HR (separate)	Model 2 HR (separate)		Model 2 HR (mutually adj.)	P (mutual)	
Excess adiposity	3.19 (2.24–4.53)	3.18 (2.24–4.52)		2.97 (2.00–4.41)	<0.001	
hypertriglyceridaemia	2.03 (1.57–2.61)	2.03 (1.58–2.62)		1.63 (1.23–2.18)	<0.001	
Low HDL-C	1.85 (1.43–2.41)	1.86 (1.43–2.42)		1.37 (1.03–1.83)	0.031	
hyperglycaemia	1.35 (1.05–1.72)	1.37 (1.07–1.75)		1.21 (0.92–1.60)	0.167	
Elevated BP	1.01 (0.78–1.30)	1.01 (0.79–1.31)		0.97 (0.73–1.29)	0.848	

Model 1: adjusted for age and sex. Model 2: additionally adjusted for smoking status (never/former/current) and drinking status (never/occasional). Panel B separate = each component in an individual model; mutually adjusted = all five components in a single model. HR, hazard ratio; CI, confidence interval.

#### Individual component contributions

When individual CM components were each evaluated in separate Cox models (Model 1: age and sex adjusted), excess adiposity showed the strongest association with incident MASLD (HR = 3.19, 95% CI: 2.24–4.53), followed by hypertriglyceridaemia (HR = 2.03, 95% CI: 1.57–2.61), low HDL-C (HR = 1.85, 95% CI: 1.43–2.41), and hyperglycaemia (HR = 1.35, 95% CI: 1.05–1.72). Elevated blood pressure was not associated with incident MASLD (HR = 1.01, 95% CI: 0.78–1.30, *P* = 0.966). All estimates were materially unchanged in Model 2 (**Table 2**). **Figure 2D** displays the mutually adjusted estimates (Model 2).

In a mutually adjusted model including all five components (Model 2), excess adiposity remained the dominant predictor (HR = 2.97, 95% CI: 2.00–4.41), followed by hypertriglyceridaemia (HR = 1.63, 95% CI: 1.23–2.18) and low HDL-C (HR = 1.37, 95% CI: 1.03–1.83). hyperglycaemia (HR = 1.21, 95% CI: 0.92–1.60) and elevated blood pressure (HR = 0.97, 95% CI: 0.73–1.29) did not reach statistical significance after mutual adjustment (**Table 2**). These mutually adjusted estimates should be interpreted as partial (conditional) effects rather than independent causal effects, given the physiological collinearity among the five CM components.

To evaluate whether the null association for elevated blood pressure reflected antihypertensive-medication confounding — a common concern in elderly populations where pharmacological blood pressure control shifts measured values in hypertensive participants toward normal — we repeated the analysis in the subset of participants not on antihypertensive medication at baseline (n = 1,588; 218 events). The age- and sex-adjusted hazard ratio for the elevated blood pressure component strengthened to HR = 1.25 (95% CI: 0.93–1.68, *P* = 0.144), and when systolic blood pressure was treated as a continuous predictor (per 10 mmHg), each 10-mmHg increment was associated with incident MASLD (HR = 1.13, 95% CI: 1.01–1.26, *P* = 0.037; **Supplementary Table 2**). These results indicate that the apparent absence of an independent blood pressure – MASLD association in the primary analysis is likely attributable, at least in part, to antihypertensive-medication confounding, and should not be interpreted as evidence against a biological role of blood pressure in MASLD.

Each additional CM component at baseline was associated with a 44% higher hazard of incident MASLD (Model 1 HR = 1.44, 95% CI: 1.31–1.59; Model 2 HR = 1.45, 95% CI: 1.32–1.60; **Table 2**). Baseline MetS conferred a twofold higher risk (Model 2 HR = 2.12, 95% CI: 1.67–2.69). Compared with the lowest TyG quartile, the highest quartile was associated with a 2.64-fold higher hazard (HR = 2.64, 95% CI: 1.84–3.79; **Supplementary Table 3**).

### Exploratory reverse cohort: association between baseline MASLD and CKM stage progression, and its dependence on baseline adiposity

**Figure 3:**
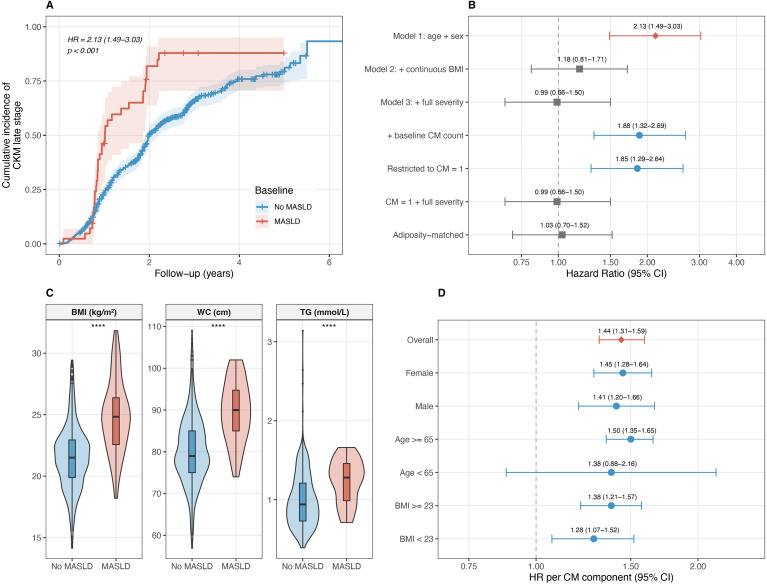
Exploratory reverse cohort and staged attenuation. **(A)** Kaplan–Meier curves for cumulative incidence of metabolic-dimension CKM late stage (≥2 CM components) by baseline MASLD status (n = 648; MASLD-positive n = 42, MASLD-negative n = 606). **(B)** Staged attenuation forest plot showing the association between baseline MASLD and CKM stage progression under progressively adjusted models. **(C)** Violin plots comparing baseline BMI, waist circumference and triglycerides between MASLD-positive and MASLD-negative participants within the CM = 1 stratum, illustrating baseline adiposity imbalance (see **Table 4**). **(D)** Forward-cohort subgroup analysis: HR per CM component for incident MASLD, stratified by sex, age and BMI (see **Supplementary Table 8**).

The exploratory reverse cohort included 648 participants at CKM early stage (0–1 CM components) at baseline; 42 (6.5%) had MASLD and 606 (93.5%) did not. Over a median follow-up of 1.77 years, 407 participants (62.8%) progressed to CKM late stage (≥2 CM components). We note that this high short-term progression rate is sensitive to measurement-day variability around CM-component thresholds and therefore likely captures both genuine early cardiometabolic deterioration and threshold-crossing noise. The cumulative incidence was 81.0% in participants with baseline MASLD and 61.6% in those without MASLD, corresponding to incidence rates of 60.23 and 32.04 per 100 person-years, respectively (**Figure 3A**).

In age- and sex-adjusted Cox regression, baseline MASLD was associated with CKM stage progression (HR 2.13, 95% CI 1.49–3.03; P < 0.001; **Table 3**). This association was substantially attenuated after adjustment for baseline BMI (HR 1.18, 95% CI 0.81–1.71; *P* = 0.398) and was no longer evident after further adjustment for continuous baseline metabolic severity markers (HR = 0.99, 95% CI 0.66–1.50; *P* = 0.969). Adjustment for baseline CM component count and restriction to participants with exactly one baseline CM component still yielded positive estimates, but the association again disappeared after adjustment for the continuous severity set within the CM = 1 stratum.

**Table 3:** Exploratory reverse cohort: staged-attenuation and baseline-comparability Cox analyses (n = 648; 407 events; median follow-up 1.77 years).

Model	Adjustment	HR (95% CI)	P value
Model 1	Age + sex	2.13 (1.49–3.03)	<0.001
Model 2	Age + sex **+ continuous BMI**	**1.18 (0.81–1.71)**	**0.398**
Model 3	Age + sex + continuous BMI, WC, FBG, TG, HDL-C, SBP (full continuous severity set)	**0.99 (0.66–1.50)**	**0.969**
Count-adjusted	Age + sex + baseline CM component count	1.88 (1.32–2.69)	<0.001
CM=1 restricted	Age + sex (n = 529)	1.85 (1.29–2.64)	<0.001
CM=1 restricted + severity	Age + sex + full continuous severity set (n = 529)	0.99 (0.66–1.50)	0.975
Adiposity-matched	Age + sex, restricted to MASLD+ and MASLD− with BMI ≥ 23 (n = 186)	1.03 (0.70–1.52)	0.864

Reverse cohort: baseline metabolic-dimension CKM early stage (0–1 CM components; MASLD group n = 42, non-MASLD group n = 606); outcome = progression to metabolic-dimension CKM late stage (≥2 CM components). The staged-attenuation sequence (Models 1 → 2 → 3) was pre-specified to quantify the contribution of baseline adiposity severity. Adjustment for continuous BMI alone (Model 2) attenuated the association to non-significance, indicating that the initial estimate is not independent of baseline adiposity. The count-adjusted, CM = 1 restricted, and adiposity-matched analyses evaluate whether baseline distance to the CKM late-stage outcome can be equalised by component-count or adiposity restrictions; these analyses illustrate that structural imbalance between MASLD-positive and MASLD-negative participants is not adequately addressed by CM count restriction alone (see **Table 4**). WC, waist circumference; FBG, fasting blood glucose; TG, triglycerides; SBP, systolic blood pressure; HR, hazard ratio; CI, confidence interval. Bold values denote the key attenuation step at which baseline-adiposity adjustment eliminates the baseline-MASLD–CKM-stage-progression association.

This attenuation was consistent with marked baseline adiposity imbalance between MASLD-positive and MASLD-negative participants. Within the CM = 1 stratum, MASLD-positive participants had higher BMI, waist circumference, and triglycerides than MASLD-negative participants (**Table 4**), and excess adiposity was more often the single qualifying CM component. In the adiposity-matched analysis restricted to MASLD-positive participants and MASLD-negative participants with BMI ≥23 kg/m², the age- and sex-adjusted estimate was null (HR = 1.03, 95% CI 0.70–1.52; *P* = 0.864). Together, these analyses indicate that the observed association is not independent of baseline adiposity and metabolic severity.

**Table 4:** Baseline imbalance between MASLD-positive and MASLD-negative participants within the CM = 1 stratum of the reverse cohort (n = 529).

Variable	MASLD+ (n = 42)	MASLD− (n = 487)	SMD
Age, years	69.7 (13.0)	69.9 (11.1)	0.01
BMI, kg/m²	24.84 (2.98)	21.89 (2.69)	1.04
Waist circumference, cm	89.6 (7.3)	81.4 (8.1)	1.07
Triglycerides, mmol/L	1.24 (0.28)	1.00 (0.35)	0.76
HDL-C, mmol/L	1.43 (0.26)	1.61 (0.53)	0.42
Systolic blood pressure, mmHg	123.9 (8.6)	128.2 (11.0)	0.43
Fasting blood glucose, mmol/L	4.75 (0.79)	4.85 (1.27)	0.09
Identity of the “single CM component”			
— Excess adiposity	81.0%	39.2%	—
— Elevated blood pressure	15.0%	49.6%	—
— hyperglycaemia/hypertriglyceridaemia/low HDL-C	4.0%	11.2%	—

Values are mean (SD) unless otherwise noted. Standardised mean differences (SMD) > 0.1 in absolute value indicate meaningful imbalance; values ≥ 0.8 are conventionally described as “large”. MASLD-positive and MASLD-negative participants within this stratum differ by approximately one full standard deviation in BMI and waist circumference, and the composition of the single CM component differs markedly between groups. This baseline structural difference accounts for the substantial attenuation of the reverse-direction hazard ratio in **Table 3**, Models 2 and 3.

### Sensitivity analyses

All primary findings were robust across sensitivity analyses. In the forward cohort, the 12-month washout analysis (excluding early events) yielded a stronger HR per CM component (1.58, 95% CI: 1.39–1.79) and a stronger CKM Stage 2 HR (3.28, 95% CI: 1.21–8.86; **Supplementary Table 4**), consistent with reduced influence of pre-existing subclinical disease. To address the definitional overlap between CM components and MASLD (which requires ≥1 CM component), we repeated the forward cohort analysis using ultrasonographic fatty liver alone (without metabolic criteria) as the outcome among participants free of fatty liver at baseline (n = 2,052; 292 events). The key estimates are reported here in the main Results text, with the full tabular results retained in **Supplementary Table 5**. All results were consistent with the primary analysis: CKM Stage 2 vs 0 HR = 2.95 (95% CI: 1.39–6.26), per-stage trend HR = 1.91 (95% CI: 1.47–2.48), and HR per CM component = 1.46 (95% CI: 1.32–1.61). In the cross-sectional analysis, using fatty liver alone as the outcome similarly yielded OR = 1.90 per component (95% CI: 1.75–2.05), close to the primary MASLD OR of 1.94 (**Supplementary Table 5**).

In the reverse cohort, the washout analysis (≥1-year follow-up) yielded HR = 2.19 (95% CI: 1.30–3.71, *P* = 0.003; **Supplementary Table 6**). Adjustment for lifestyle factors gave HR = 2.14 (95% CI: 1.50–3.05; **Supplementary Table 6**). The proportional hazards assumption was met for all models (Schoenfeld global P > 0.20; **Supplementary Table 7**).

In a complete-case analysis restricted to participants with all five CM components assessable (n = 1,623; 79% of forward cohort), the HR per CM component was 1.47 (95% CI: 1.32–1.63), consistent with the primary estimate of 1.44.

Subgroup analyses of the forward cohort showed consistent effect directions: female HR = 1.45 (95% CI: 1.28–1.64), male HR = 1.41 (95% CI: 1.20–1.66); age ≥65 HR = 1.50 (95% CI: 1.35–1.65); BMI <23 HR = 1.28 (95% CI: 1.07–1.52), BMI ≥23 HR = 1.38 (95% CI: 1.21–1.57; **Supplementary Table 8**).

## Discussion

This community-based longitudinal study provides three main findings regarding the relationship between the metabolic-dimension CKM staging system and MASLD. First, metabolic-dimension CKM Stage 2 (≥2 metabolic risk factors) was associated with substantially higher incident MASLD risk (HR = 2.33), with a dose–response gradient across stages (HR = 1.80 per stage). Second, component-level analysis identified excess adiposity (mutually adjusted HR = 2.97) and hypertriglyceridaemia (HR = 1.63) as the dominant predictors of incident MASLD; the apparent absence of an independent association for elevated blood pressure in the primary analysis was attributable to antihypertensive-medication confounding, and a positive association emerged in the medication-naive subset (each 10-mmHg increment in systolic blood pressure: HR = 1.13, *P* = 0.037). Third, in an exploratory reverse analysis, an age- and sex-adjusted association between baseline MASLD and CKM stage progression (HR = 2.13) was not independent of baseline adiposity: the estimate attenuated to HR = 1.18 after adjustment for continuous BMI alone, and to HR = 0.99 after adjustment for the full continuous severity set. In this dataset we therefore found no evidence of an association between baseline MASLD and short-term CKM progression that is independent of baseline metabolic severity.

The metabolic-dimension CKM staging analysis showed that the risk of incident MASLD increased substantially at Stage 2, with a significant per-stage trend (HR = 1.80 per stage). Although the comparison between Stage 1 and Stage 0 did not reach statistical significance (HR 1.14, 95% CI 0.56–2.35), the wide confidence interval—stemming from the limited size of the Stage 0 reference group (n = 119, 9 events) —does not rule out clinically meaningful hazards. Our findings are consistent with the rationale for CKM staging, in which clustering of metabolic risk factors confers risk that increases monotonically across stages (6). Zheng et al. (12) reported progressive increases in hepatic steatosis prevalence across CKM stages in a Chinese population; our prospective data extend their cross-sectional findings by demonstrating that metabolic-dimension CKM staging predicts future MASLD development, supporting its use as a risk stratification tool for hepatic screening in community settings. Our operationalisation was restricted to the metabolic domain because routine community health examinations did not include subclinical cardiovascular or kidney imaging; this is a substantial deviation from the full AHA CKM framework (which includes Stages 3–4 for subclinical and clinical CVD/CKD) and we made this restriction explicit in both the title and throughout the manuscript.

The component-level analysis refines prior observations. Excess adiposity emerged as the strongest predictor (mutually adjusted HR = 2.97), with an effect size substantially exceeding that of dyslipidaemia or hyperglycaemia, consistent with the established role of adipose tissue dysfunction in MASLD — visceral adiposity increases hepatic free fatty acid delivery and *de novo* lipogenesis while promoting adipose inflammation (7, 8). hypertriglyceridaemia (HR = 1.63) and low HDL-C (HR = 1.37) also showed component-level associations in mutually adjusted models, consistent with their role as markers of insulin resistance and hepatic lipid overload. These mutually adjusted estimates should be interpreted as partial (conditional) rather than independent causal effects, given the physiological collinearity among the five CM components.

The apparent null association for elevated blood pressure in the primary mutually adjusted model (HR = 0.97) should not be interpreted as evidence against a biological role of blood pressure in MASLD, since hypertension and steatotic liver disease are closely linked in cardiometabolic disease pathways (19). Pharmacological blood pressure control is widespread in elderly community populations, and antihypertensive therapy shifts measured systolic and diastolic blood pressure in hypertensive participants toward normal, biasing the exposure classification toward the null. Consistent with this explanation, excluding participants on antihypertensive medication strengthened the age- and sex-adjusted component-level HR (to 1.25) and — more informatively — yielded a positive, statistically significant association for continuous systolic blood pressure (HR = 1.13 per 10 mmHg, *P* = 0.037) in the medication-naive subset. Comprehensive lipid-lowering medication data were not available in this cohort, so analogous analyses for the hypertriglyceridaemia and low HDL-C components could not be performed; residual medication-related confounding for the lipid components cannot be excluded.

The reverse analysis showed a different pattern from the primary forward cohort. The age- and sex-adjusted association between baseline MASLD and CKM stage progression (HR = 2.13) was substantially attenuated after adjustment for continuous BMI (HR = 1.18) and was no longer evident after adjustment for the full continuous severity set (HR = 0.99). This pattern is consistent with marked baseline adiposity imbalance between MASLD-positive and MASLD-negative participants, including within the CM = 1 stratum, where BMI differed by approximately one standard deviation and excess adiposity was much more common as the single qualifying CM component. In the adiposity-matched analysis, the estimate was null (HR = 1.03). Thus, the reverse-direction findings should be viewed as exploratory and do not show an association independent of baseline adiposity and related metabolic severity. Longer follow-up with repeated measurements will be needed to determine whether hepatic steatosis contributes to cardiometabolic progression beyond shared adiposity-related risk.

MASLD requires at least one cardiometabolic risk component, and metabolic-dimension CKM staging is based on the same component set. Therefore, part of the association between CKM stage and MASLD could reflect shared definitions, particularly in the cross-sectional analysis where MASLD is absent at zero CM components by definition. We therefore repeated the forward-cohort analyses using ultrasonographic fatty liver alone, without requiring metabolic criteria, as a sensitivity outcome. The estimates were consistent with the primary MASLD outcome: Stage 2 versus Stage 0 HR 2.95 versus 2.33, and per-component HR 1.46 versus 1.44. The cross-sectional OR per CM component was also similar for fatty liver alone and MASLD (1.90 versus 1.94; **Supplementary Table 5**). These results suggest that the forward-direction findings were not solely explained by shared component definitions.

Our forward-cohort findings are consistent with, and extend, a growing literature on CKM syndrome and liver disease. Chen et al. (10) found that advanced CKM staging predicted mortality among MASLD patients. Zhou et al. (11) showed associations between CKM syndrome and liver fibrosis risk. Hu et al. (20) reported that MASLD was associated with advanced CKM syndrome. Liang et al. (21) demonstrated in the UK Biobank that CKM progression predicted MASLD hospitalisation; our study complements their findings by using community-based ultrasonographic screening, which captures early-stage MASLD, and by providing component-level granularity under mutual adjustment. Liu et al. (22) reported that NAFLD was associated with CKM progression; our staged-attenuation analysis suggests caution when interpreting such reverse-direction estimates, since initial association in our cohort was attenuated by baseline adiposity imbalance. The proposals by Zhou et al. (9), Bian et al. (23), and Zannad et al. (24) to expand CKM to include hepatic endpoints are supported by our forward-direction data, which show robust prospective associations between metabolic-dimension CKM staging/components and incident MASLD.

This study has several limitations. First, CKM staging was operationalised using the metabolic risk dimension only. Subclinical and clinical cardiovascular and kidney disease components were not available in the routine community health examination data, so our findings should not be interpreted as applying to the full AHA CKM staging framework. Second, the exploratory reverse analysis was limited by the small number of MASLD-positive participants at baseline and by marked baseline adiposity imbalance; after adjustment for continuous BMI and related metabolic severity, the association was no longer evident. The reverse-direction findings should therefore be regarded as exploratory and do not establish an association independent of baseline adiposity. Third, hepatic steatosis was assessed by B-mode ultrasonography without severity grading, and this modality cannot distinguish steatohepatitis from simple steatosis. Fourth, the cohort was predominantly elderly and metabolically high-risk, with most participants already classified as metabolic-dimension CKM Stage 2 at baseline and a small Stage 0 reference group; generalisation to younger or metabolically healthier populations requires caution. The relatively short follow-up also limits inference about longer-term cardiometabolic trajectories. Finally, medication data were incomplete, lipid-lowering therapy was not systematically recorded, death could not be modelled as a competing risk, and residual confounding from lifestyle, dietary, environmental, or other unmeasured factors cannot be excluded.

## Conclusion

In this elderly community cohort from southern China, metabolic-dimension CKM staging predicted incident MASLD with a dose–response gradient, and Stage 2 (≥2 metabolic risk factors) was associated with significantly elevated risk (HR = 2.33 in primary analysis and HR = 2.95 with fatty-liver-alone sensitivity outcome). Excess adiposity and hypertriglyceridaemia were the dominant component-level predictors. The apparent null association for elevated blood pressure in the primary mutually adjusted model was likely attributable to antihypertensive-medication confounding; in the medication-naive subset, each 10-mmHg increment in systolic blood pressure was associated with incident MASLD. In the exploratory reverse analysis, we did not find evidence that baseline MASLD was associated with CKM stage progression after accounting for baseline adiposity. Our forward-direction findings support prospective evaluation of hepatic assessment within extensions of the CKM framework; however, whether hepatic steatosis makes an independent contribution to cardiometabolic trajectory beyond shared adiposity-driven insulin resistance cannot be established from the current data and will require studies with longer follow-up, repeated measurement schemes, and populations in which MASLD status is not so tightly tied to baseline adiposity.

## Data Availability

The raw data supporting the conclusions of this article will be made available by the authors, without undue reservation.
